# Low-Power OR Logic Ferroelectric In-Situ Transistor Based on a CuInP_2_S_6_/MoS_2_ Van Der Waals Heterojunction

**DOI:** 10.3390/nano11081971

**Published:** 2021-07-31

**Authors:** Kun Yang, Shulong Wang, Tao Han, Hongxia Liu

**Affiliations:** Key Laboratory for Wide-Band Gap Semiconductor Materials and Devices of Education, The School of Microelectronics, Xidian University, Xi’an 710071, China; kuny2019@163.com (K.Y.); slwang@xidian.edu.cn (S.W.); taohan373@gmail.com (T.H.)

**Keywords:** two-dimensional materials, heterojunction, ferroelectric, OR logic

## Abstract

Due to the limitations of thermodynamics, the Boltzmann distribution of electrons hinders the further reduction of the power consumption of field-effect transistors. However, with the emergence of ferroelectric materials, this problem is expected to be solved. Herein, we demonstrate an OR logic ferroelectric in-situ transistor based on a CIPS/MoS_2_ Van der Waals heterojunction. Utilizing the electric field amplification of ferroelectric materials, the CIPS/MoS_2_ vdW ferroelectric transistor offers an average subthreshold swing (SS) of 52 mV/dec over three orders of magnitude, and a minimum SS of 40 mV/dec, which breaks the Boltzmann limit at room temperature. The dual-gated ferroelectric in-situ transistor exhibits excellent OR logic operation with a supply voltage of less than 1 V. The results indicate that the CIPS/MoS_2_ vdW ferroelectric transistor has great potential in ultra-low-power applications due to its in-situ construction, steep-slope subthreshold swing and low supply voltage.

## 1. Introduction

With the feature size of transistors scaling down, the emergence of various parasitic effects severely restricts the development of Moore’s Law, among which short-channel effects play a key role [1]. The derivation of two-dimensional materials means that this parasitic effect is expected to be solved [2,3]. Owing to the strong covalent bond between metal and chalcogenide atoms, even if the thickness is reduced to the atomic level, the material’s characteristics can be completely retained [4]. It has been proven that two-dimensional (2D) transition metal dichalcogenides (TMDs) have a natural immunity to short channel effects [5,6]. In addition, the Boltzmann distribution of the electron at room temperature hinders the optimization of the switching characteristics of the metal oxide field-effect transistor, which sets a barrier for the power consumption reduction. The ferroelectric field-effect transistor provides new strategies for breaking the thermodynamics limit [7,8]. For digital circuits based on CMOS cells, the most important factor restricting the reduction in the size of the integrated circuits is the contradiction between performance improvement and power consumption [9]. Researchers use the unique residual polarization characteristics of ferroelectric materials to prepare a variety of transistor-like structures with sub-threshold swings less than 60 mV/dec, which greatly promotes the computing speed of chips and the reduction of power consumption [10,11,12,13]. Not only that, thanks to the strong photogating effect induced by the ferroelectric local electrostatic field, ferroelectric materials are widely used in the field of photoelectric detection [14,15,16]. Ferroelectric materials, as a general term describing the characteristics of materials with polarization that can be reversed under an applied electric field, exhibit stable states of collectively ordered electrical dipoles [17,18]. Ferroelectric materials have a wide variety of types, such as organic ferroelectric polymer P(VDF-TrFE) [19] and inorganic ferroelectric oxide-fluorite-structure binary oxides (fluorites), i.e., doped-HfO_2_ [20], Van der waal ferroelectric materials [21]. The huge material manifestation greatly expands the application fields of ferroelectric materials, such as flexible electronics, non-volatile memory and so on [22,23]. With the rapid development of the field of artificial intelligence, the demand for hardware computing capabilities is increasing dramatically, which has promoted the derivation of new types of storage devices. Among these nonvolatile memory technologies, the FeFET—as a new transistor type memory architecture—gradually appeared in the public eye. The natural capacitance mismatch of ferroelectric materials results in a larger storage window, which promotes the reduction of the data retention and the erasing of the voltage. Brain-like synaptic devices made of ferroelectric materials can realize the basic unit of neuromorphic computing, and the constructed neural network unit has a high degree of parallelism [15,24,25].

Compared with traditional ferroelectric materials which are epitaxially grown, van der Waals heterojunctions exhibit excellent innate properties in the improvements of interface defects [26]. Due to the lack of dangling bonds on the surface of the two-dimensional material, it will become difficult to use epitaxial high-quality ferroelectric materials on the surface of the two-dimensional material through the atomic layer deposition (ALD) process [27]. With the emergence of two-dimensional dielectric materials, van der Waals heterojunctions open the pathway for the integration of 2D semiconductors with dielectrics [28]. Recently, the ferroelectricity of some two-dimensional layered materials was confirmed successively by experiments. Choosing the appropriate 2D ferroelectric dielectric is vital, given that it directly determines the performance of the device [29]. The candidate needs to maintain stable ferroelectricity at room temperature when the channel body is reduced to nanometers. The stable switchable polarization in CuInP_2_S_6_ (CIPS) at room temperature has been experimentally observed even at scales down to 4 nm, which exhibits its potential as a 2D ferroelectric material [21,30].

In this work, we demonstrate the ferroelectric in-situ transistor based on CIPS/MoS_2_, with CIPS and MoS_2_ as the ferroelectric dielectric and channel semiconductor, respectively. The CIPS/MoS_2_ vdW ferroelectric FET exhibits a steep subthreshold swing (SS) of 52 mV/dec over three decades of drain current. The in-situ OR logic transistor is built under the combined effect of the top and back gate. OR logic operation in single-channel transistors is successfully achieved, and the excellent device performance is demonstrated by a steep switch characteristic and low supply voltage. The proposal of the CIPS/MoS_2_ OR logic ferroelectric transistor provides a new idea for the construction of low-power complex-logic transistors.

## 2. Experimental Details

### 2.1. Fabrication of the CIPS/MoS_2_ vdW Ferroelectric Transistor

The highly p-doped silicon substrates with the resistivity of 0.008 Ω·cm^−1^ were cleaned by a standard RCA process, and all of the samples were dried with high-purity N_2_. The native SiO_2_ on the substrate was removed by immersion in diluted HF solution (4%) for 60 s before the deposition of the HfZrO_x_ dielectric. Then, the HfZrO_x_ gate dielectric was deposited by the atomic layer deposition reactor (Picosun R-150) with tetradiethylamino zirconium, tetradimethylamino hafnium as the precursor source, and H_2_O as the oxidant. The thickness of the HfZrO_x_ film was measured by the spectroscopic ellipsometry (SE) system (J.A. Woollam Co. M2000U, Lincoln, NE, USA). Then, the MoS_2_ flake was micromechanically exfoliated onto the HfZrO_x_ substrates. The CIPS was micromechanically exfoliated on polydimethylsiloxane held on a glass slide. The heterostructures of the CIPS/MoS_2_ were fabricated using dry transfer, as reported [31]. The electrodes were formed by a standard e-beam lithography process, Cr/Au (10 nm/50 nm) deposited by thermal evaporation, and a lift-off process. Finally, the device was annealed at 300 °C for 2 h under an Ar atmosphere to form ohmic contact.

### 2.2. Characterization Method

The thickness of the CIPS and MoS_2_ were measured using an atomic force microscope system (BRUKER Multimode 8, Berlin, Germany). Raman spectra measurements was carried out on a Horiba LabRAM HR800 Raman spectrometer (Paris, France) with a laser of a 532 nm wavelength. The electric characteristics of the CIPS/MoS_2_ vdW ferroelectric transistor were measured using an Agilent B1500A analyzer (Santa Clara, CA, USA). The measurement data was collected using a cascade probe station at room temperature.

## 3. Results and Discussion

### 3.1. Material Characteristics and Electrical Performance of the CIPS/MoS_2_ vdW Ferroelectric Transistor

**Figure 1:**
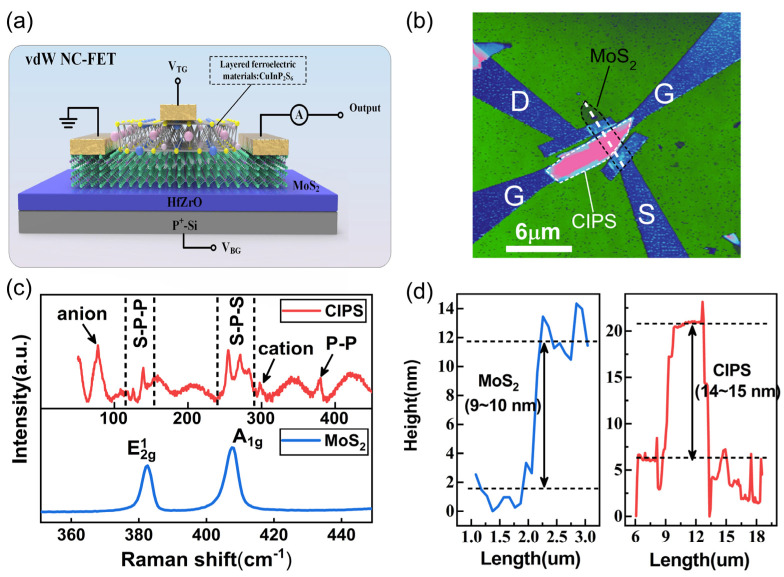
(**a**) The schematic diagram of the CIPS/MoS_2_ vdW in-situ ferroelectric transistor. (**b**) The surface morphology of the CPIS/MoS_2_ ferroelectric transistor, as measured by AFM. (**c**) The Raman spectrum of the exfoliated CIPS and MoS_2_ flake. (**d**) The height distribution along the white dotted line in the AFM image.

Figure 1a shows the schematic structure of the CPIS/MoS_2_ vdW double-gate ferroelectric transistor, consisting of a multilayers MoS_2_ channel material, and CIPS and HfZrO_x_ as the top and back dielectric, respectively. The heavily doped silicon substrate serves as the back gate. The surface morphology of the CPIS/MoS_2_ ferroelectric transistor is shown in Figure 1b, as measured by the atomic force microscope (AFM), where the gate length is slightly shorter than the channel. Then, the Raman spectrum of CIPS and MoS_2_ are illustrated in Figure 1c in order to identify the material properties, exhibiting a similar ferroelectric phase consistent with the reported bulk CIPS crystals and multilayer MoS_2_ Raman characteristics [29,32,33]. Figure 1d shows the height distribution along the white dotted line in Figure 1b. The thickness of the MoS_2_ is approximately 9 nm, which guarantees the high performance and reliability of the device. The monolayer MoS_2_ film is easily disturbed by external factors, exhibiting low conductivity, high contact resistance and poor reliability. The literatures indicates that the MoS_2_ transistor with multilayers exhibits the best device performance [33,34]. The appropriate CIPS (14~15 nm) can reinforce the intensity of the polarization in order to obtain the ideal subthreshold swing (SS).

**Figure 2:**
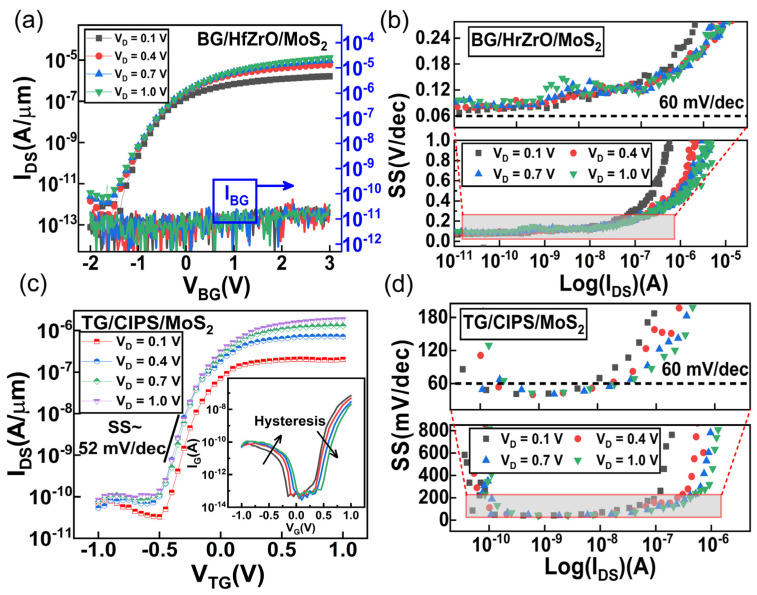
The electric characteristics of the CIPS/MoS_2_ vdW ferroelectric transistor at room temperature. (**a**,**b**) The I-V characteristic and the SS-I_DS_ characteristic of the back-gate configuration, respectively. (**c**,**d**) The I-V characteristic and the SS-I_DS_ characteristic of the top-gate configuration, respectively.

Figure 2 shows the electrical performance of the top-gate transistor and the back-gate transistor, respectively. The transfer characteristic of the back-gate transistor is shown in Figure 2a with the heavily doped silicon substrate as the back gate, and with the top gate floating. The I_DS_-V_BG_ characteristic shows a typical n-type behavior. The on/off ratio can reach 10^7^ when the V_BG_ ranges from −2 V to 3 V. As the drain voltage increases, the I_DS_-V_BG_ of the back-gate transistor begins to drift negatively, resulting in a drain-induced barrier reduction effect [35]. At the same time, it should be noticed that the sub-threshold swing exhibits a mild degradation compared to V_D_ = 0.1 V, which is because the trap capture is enhanced at a high drain voltage [36]. The gate leakage current remains consistent across the entire voltage range thanks to the dense HfZrO_x_ oxide. According to the following formula [37]:

(1) $$SS=\partial V_{BG}/\partial \left(\log I_{DS}\right)$$

the SS of back gate MoS_2_ MOSFET is extracted, as shown in Figure 2b. The minimum SS is derived to be 67 mV/dec for V_D_ = 0.1 V and 79 mV/dec for V_D_ = 1 V. The minimum SS increases to a greater extent as the drain voltage increases, and both values are above the thermionic limit (~60 mV/dec) due to the Boltzmann distribution of electrons at room temperature. On the contrary, the top gate MoS_2_ shows an outstanding switching characteristic. It is well known that CIPS has ferroelectric properties, as has been reported [29]. The polarization generated by the ferroelectric material causes the gate electric field to be locally amplified so that a sustained sub−60 mV/dec switching can be obtained. The electric characteristic of the so-called CIPS/MoS_2_ vdW ferroelectric transistor is illustrated in Figure 2c. The I_DS_-V_TG_ characteristic shows a preferable consistency at different drain voltages. Similar to the back-gate transfer characteristic, the drain-induced barrier reduction effect is weak except for V_D_ = 0.1 V. The charge trapping at the interface of MoS_2_/dielectric is not only affected by the gate voltage, but is also modulated by the drain voltage [36]. Therefore, when the drain voltage is low, the charge trapping increases and the threshold voltage shifts in the positive direction. From Figure 2c, the impact of a negative capacitance (NC) effect in vdW CIPS/MoS_2_ FET is revealed by an observed conversion of the gate leakage current at V_TG_ = 0 V, which is a unique trait and not seen in conventional gate dielectric. This phenomenon is the result of the combined effect of the polarization electric field and the drain-induced-barrier-rising effect of NC [20]. Despite the incomplete gated channel limits in the on-state current, the SS extracted from the top–gate transfer curve is below the thermionic limit by three orders of magnitude, as illustrated in Figure 2d. The CIPS/MoS_2_ vdW ferroelectric transistor exhibits an average SS of 52 mV/dec, and a minimum SS of 40 mV/dec.

### 3.2. The Performance Optimization of the CIPS/MoS_2_ vdW Ferroelectric Transistor

**Figure 3:**
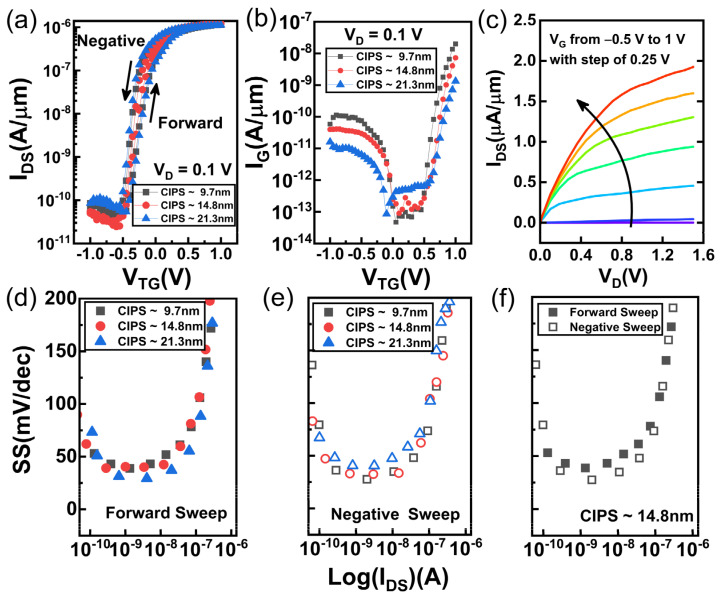
(**a**) The dual-direction transfer characteristic of CIPS/MoS_2_ NC-FET with 9.7 nm, 14.8 nm and 21.3 nm CIPS. (**b**) The gate leakage current of CIPS/MoS_2_ NC-FET with 9.7 nm, 14.8 nm and 21.3 nm CIPS. (**c**) The output characteristic of vdW NC-FET with a CIPS of 14.8 nm. (**d,e**) The subthreshold swing as functions of log(I_DS_) under forward sweep and negative sweep, respectively. (**f**) The subthreshold swing distribution of vdW NC-FET with a CIPS of 14.8 nm under dual-direction sweeping.

The hysteretic characteristic seriously affects the stability of the NC-FET device due to the capacitance mismatch [38,39,40]. Stable switching can be achieved by matching the appropriate negative capacitance, and the degree of capacitance matching will directly affect the electrical performance of the NC-FET [41,42,43]. The essence of capacitance matching is the coupling relationship between the negative gate capacitance |C_FE_| and the gate capacitance C_MOS_ [44]. Because C_MOS_ depends on the channel materials, channel thickness and other factors, we mainly adjust |C_FE_| to make the CIPS/MoS_2_ NC-FET achieve an ideal capacitance matching relationship. Theoretical research shows that |C_FE_| in NC-FET can be approximated as [45]:

(2) $$\left|C_{\mathrm{FE}}\right|=A_{\mathrm{FE}}\cdot \frac{2}{3\sqrt{3}}\cdot \frac{P_{r}}{E_{C}\cdot t_{\mathrm{FE}}}$$

where A_FE_, E_C_, Pr, t_FE_ represent the area of the ferroelectric dielectric, coercive electric field strength, residual polarization charge density and ferroelectric film thickness, respectively. Unlike traditional Hf-based ferroelectric materials, the ferroelectricity of a two-dimensional material is the product of its own lattice structure, and does not rely on the recrystallization caused by the annealing effect [21]. Therefore, for the same two-dimensional ferroelectric material, the coercive electric field intensity and the remnant polarization charge density are constants, and |C_FE_| mainly depends on the thickness of the ferroelectric film. Herein, the CIPS/MoS_2_ NC-FET’s performance optimization is achieved by adjusting the ferroelectric film thickness. Figure 3a shows the dual-direction transfer characteristic of CIPS/MoS_2_ NC-FET with 9.7 nm, 14.8 nm and 21.3 nm CIPS. The minimum hysteresis window of 53.4 mV at I_D_ = 1 nA is obtained for ~14.8 nm CIPS less than the 131 mV for ~9.7 nm and 171.8 mV for ~21.3 nm. At the same time, the gate leakage is effectively suppressed for ~14.8 nm CIPS due to better capacitance matching, as shown in Figure 3b. Figure 3c shows the I_D_-V_DS_ characteristic of vdW ferroelectric FET with ~14.8 nm CIPS under V_GS_ from −0.5 V to 1 V, with a step of 0.25 V. The vdW ferroelectric FET has the current density of 2 µA/µm at a low supply voltage (1.2 V), which can greatly reduce the switching power consumption of the transistor according to the following formula [46]:

(3) $$P_{\mathrm{swtiching}}=fCV_{\mathrm{DD}}^{2}$$

where f is the clock frequency, C is the total output capacitance and V_DD_ is the supply voltage. Figure 3d,e show the subthreshold swing of three different thickness devices under forward sweep and negative sweep, respectively. As the thickness of the CIPS increases, the positive subthreshold swing decreases slightly, and an increased switching speed of the device is obtained. The negative transfer characteristic curve tends to recover, which is related to the inversion of the polarization electric field. In order to better demonstrate the correlation between the device performance and the sweep direction, Figure 3f shows the subthreshold swing distribution under dual-direction sweeping, from which sustained sub−60 mV/decade switching and stable device repeatability is obtained.

### 3.3. The Demonstration of the OR Logic Function

**Figure 4:**
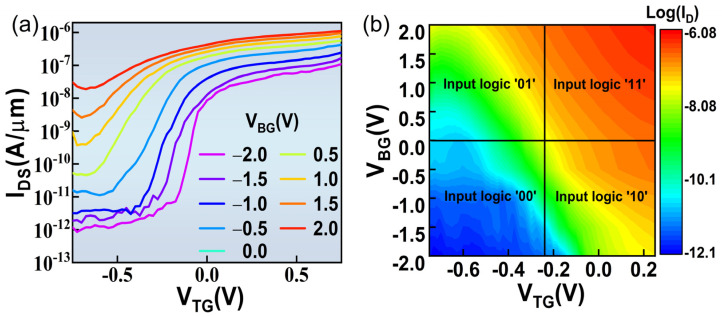
(**a**) The electric characteristic of CIPS/MoS_2_ vdW in-situ OR-Logic operation; the inset shows the measurements configuration of the CIPS/MoS_2_ vdW in-situ OR-Logic transistor. (**b**) The 2D mapping image of the output signals log(I_DS_) as functions of the input signals V_TG_ and V_BG_.

In order to prevent the incomplete-gated channel, a dual-gated operation of the MoS_2_ transistor is conducted. Under the blessing of the back-gate electric field, the double-gate transistor exhibits better switching characteristics, thanks to the better electrostatic control over the channel. The principle is similar to Fin-FET and a gate-all-around (GAA) transistor; however, the difference is that a dual-gate transistor provides two independent gate controls. By modulating the electrostatic intensity of the top gate and the back gate, the I_DS_ and V_th_ can be continuously adjusted. The I-V characteristic of the dual-gated transistor is illustrated in Figure 4a at V_D_ = 0.1 V, with V_TG_ sweeping uninterruptedly from −0.75 V to 0.75 V and V_BG_ keeping a constant voltage from −2 V to 2 V, with a step of 0.5 V. As the back gate voltage V_BG_ increases, the behavior of the dual-gated transistor gradually changes from the switching characteristics of field-effect transistors to a resistance-like normally-on state uncontrolled by the top gate, which indicates that the back and top gate can work with each other well [47]. Due to the existence of the back-channel, the on-state current increased by 10 times compared to the single top-gate transistor. At the same time, under the action of the bidirectional electric field, the entire channel is depleted, and the off-state current is reduced by two orders of magnitude. The OR logic operation is the basic logic unit in digital circuits, and the OR logic operation was implemented through in situ dual-gate architecture [33,48]. Next, we demonstrated the OR logic function of the CIPS/MoS_2_ vdW ferroelectric transistor, in which the top-gate and back-gate electrodes serve as two signal input terminals, respectively. In order to expressly show the OR logic function of the dual-gated MoS_2_ transistor with the CIPS/MoS_2_/HfZrO_x_ structure, the 2D mapping image of log(I_DS_) as functions of the input signals V_TG_ and V_BG_ is exhibited in Figure 4b. The output signals log(I_DS_) are poor when the input signal are in a ‘00′ state, as shown in blue region of Figure 4b. As the conversion of input signals, the output signals are reinforced obviously, especially when the input signal of an ‘11′ state is applied. By regulating the gate input signal, the OR logic operation can be realized in a single channel MoS_2_ transistor, such that the complexity of the digital circuits can be effectively reduced. More importantly, the power consumption of the OR logic CIPS/MoS_2_ vdW ferroelectric transistor is limited due to the low leakage current, small input voltage and steep subthreshold swing.

### 3.4. The Discussion of the Electronic Transport Mechanism

**Figure 5:**
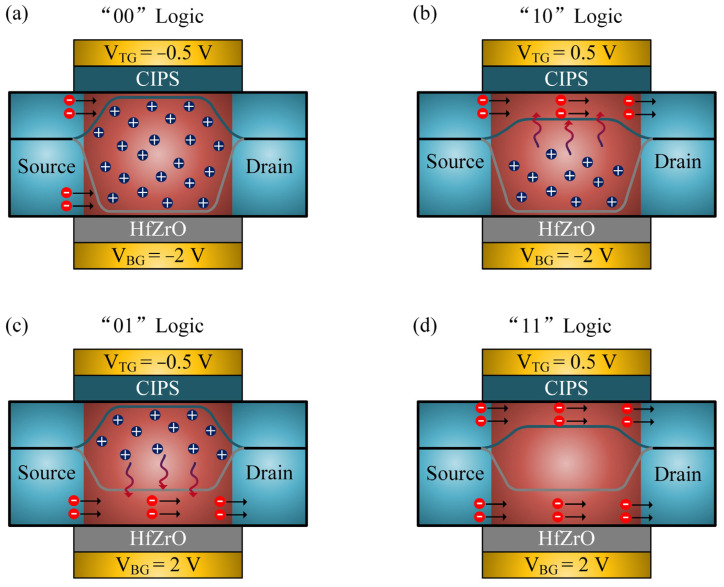
Schematic illustrations of the OR-Logic operation mechanism of the CIPS/MoS_2_ vdW in-situ OR-Logic transistor. (**a**) “00” logic operation. (**b**) “10” logic operation. (**c**) “01” logic operation. (**d**) “11” logic operation.

The metal oxide semiconductor field-effect transistor is a voltage-type control device, the conduction characteristic of which depends entirely on whether the electrostatic induction channel is formed [49]. The conduction state of the transistor is controlled by the barrier height between the source and drain. For dual-gated transistors, the barrier height on both sides of the channel determines the conduction current of the device. In order to better understand the conduction mechanism, the intuitive understanding can be obtained from the energy band diagram. Figure 5 illustrates the ways in which the barrier height changes for different input signals. As shown in Figure 5a, when V_TG_ = −0.5 V, V_BG_ = −2 V is applied, and the barrier of the top and back channel is formed simultaneously, inducing fewer electrons to move from the source to the drain. Thus, the output logic of the device is the ‘0′ state. When the “01” or “10” logic input signal is applied to the two gates, the barrier of the MoS_2_ interface drops such that the top or back electron channel is formed, as shown in Figure 5b,c. Compared with the “00” input signals, the on-state current is enhanced due to the single electron channel formed. However, because the one channel is in an accumulation state, the Coulomb scattering in another channel is enhanced because of the coupling of the top and back channel, as shown in Figure 5b,c, meaning that the output current is partly limited [50]. Only when the “11” input signal is applied at the two gates are the top and back inversion channel formed simultaneous, as shown in Figure 5d, causing the conductance between the source and drain to increase. In this case, the output current rises in comparison to the “10” and “01” logic input signals. The logic operation indicates that OR logic computing can be achieved in a single 2D vdW transistor. Compared with silicon-based field-effect transistors, the large surface area-to-volume ratio of 2D materials enables the construction of multiple logic gates in single 2D transistors, which greatly improves the area-efficiency of chips and promotes power consumption reduction.

## 4. Conclusions

In summary, we have fabricated a CIPS/MoS_2_ van der Waals ferroelectric transistor, where CIPS and MoS_2_ serve as the ferroelectric dielectric and channel materials, respectively. Benefitting from the smooth and clean interface of CIPS/MoS_2_, the vdw NC-FET shows high switch performance. Sustained sub−60 mV/dec SS switching is obtained under the effect of ferroelectric materials. Furthermore, we demonstrate the OR logic computing of the CIPS/MoS_2_/HfZrO_x_ dual-gated transistor. Only when the “00” logic input signal is applied at the two gates is the dual-gated transistor in the off state, which originates from the independent adjustment of the barrier height. What is more, the fabricated OR logic vdw ferroelectric transistor with an in-situ construction exhibits a satisfactory device performance with steep switch characteristics and a low supply voltage. This successful demonstration for the integration of a vdW ferroelectric dielectric and semiconductor provides a strategy to improve the area-efficiency of chips, and to meet the low-power demand in the post-Moore period.

## Data Availability

The data is available from the corresponding authors upon reasonable request.
